# The antipsychotic drug sulpiride in the ventral pallidum paradoxically impairs learning and induces place preference

**DOI:** 10.1038/s41598-022-23450-z

**Published:** 2022-11-10

**Authors:** Daniella Dusa, Tamás Ollmann, Veronika Kállai, László Lénárd, Erika Kertes, Beáta Berta, Ádám Szabó, Kristóf László, Rita Gálosi, Olga Zagoracz, Zoltán Karádi, László Péczely

**Affiliations:** 1 Learning in Biological and Artificial Systems Research Group, Institute of Physiology, Medical School, University of Pécs, Pécs, Hungary; 2 Neuropeptides, Cognition, Animal Models of Neuropsychiatric Disorders Research Group, Institute of Physiology, Medical School, University of Pécs, Pécs, Hungary; 3 Institute of Physiology, Medical School, University of Pécs, Szigeti Str. 12, P.O. Box: 99, Pécs, 7602 Hungary; 4 Molecular Neuroendocrinology and Neurophysiology Research Group, Szentágothai Research Centre, University of Pécs, Pécs, Hungary; 5 Centre for Neuroscience, University of Pécs, Pécs, Hungary

**Keywords:** Neuroscience, Learning and memory, Motivation, Reward

## Abstract

Sulpiride, as a D2-like dopamine (DA) receptor (D2R) antagonist, is an important antipsychotic drug in the treatment of schizophrenia. Recently, we have shown that the activation of D2Rs in the ventral pallidum (VP) modulates the activity of the ventral tegmental area (VTA) DAergic neurons. According to our hypothesis, intra-VP sulpiride can influence the motivational and learning processes, pervasively modifying the behavior of examined animals. In the present study, sulpiride was microinjected into the VP of male Wistar rats in three different doses. Morris water maze (MWM) test was applied to investigate the effects of sulpiride on spatial learning, while conditioned place preference (CPP) test was used to examine the potential rewarding effect of the drug. In order to show, whether the animals can associate the rewarding effect with an area which can be recognized only on its spatial location, we introduced a modified version of the CPP paradigm, the spatial CPP test. Our results show that the intra-VP sulpiride dose-dependently impairs learning processes. However, the largest dose of sulpiride induces place preference. Results of the spatial CPP paradigm demonstrate that the animals cannot associate the rewarding effect of the drug with the conditioning area based on its spatial location. In the CPP paradigm, locomotor activity decrease could be observed in the sulpiride-treated rats, likely because of a faster habituation with the conditioning environment. In summary, we can conclude that intra-VP sulpiride has a dual effect: it diminishes the hippocampus-dependent spatial learning processes, in addition, it has a dose-dependent rewarding effect.

## Introduction

The ventral pallidum (VP) is a basal forebrain limbic structure. Recently, it has become the focus of attention, since VP demonstrates relative reward value earlier than the nucleus accumbens (NAC)^1^, and a subset of VP neurons encodes reward prediction error more robustly than the NAC^2^. Furthermore, VP neurons respond not only to reward but also to punishment and outcome-predicting stimuli^3^.

The VP is innervated by the ventral tegmental area (VTA) DAergic fibers^4^, and it has been shown that the hippocampus, brain structure involved in spatial orientation and learning processes^5^ controls entry of information into long-term memory via the NAC-VP-VTA axis^6^. The D2-like DA receptors (D2R) can be found in the VP^7^. The indirect DA agonist psychostimulant drugs, namely cocaine and amphetamine, exert their addictive effects in part via the VP^8–11^. We have demonstrated that the D2R agonist quinpirole microinjected into the VP facilitates memory consolidation in spatial learning^12^ and inhibitory avoidance learning^13^. Moreover, we have revealed in our recent study that intra-VP quinpirole induces conditioned place aversion (CPA) and decreases the activity of the VTA DAergic neurons^14^.

The D2R antagonist sulpiride is an atypical antipsychotic drug, which is used mainly in psychosis associated with schizophrenia, but it can be applied in major depressive disorder as well, and in the treatment of anxiety and mild depression^15–18^. In addition, as an antagonist of the D3 subtype of D2Rs, application of sulpiride can be a promising in addiction treatment^19–21^.

Based on the aforementioned findings, we hypothesize that sulpiride microinjected into the VP can induce conditioned place preference (CPP), in addition, it can impair learning processes. Nevertheless, as we postulated in our recent paper, induction of the CPP requires more necessary conditions, inter alia intact learning processes^22^. Since we assume that this latter can be impaired by the intra-VP sulpiride treatment, this means, that our present hypothesis includes an apparent paradox.

In order to confirm our hypothesis, in the present experiments, we used the Morris water maze (MWM) paradigm to investigate the effect of sulpiride on spatial learning, while the CPP paradigm was applied to test the potential rewarding effect of it. In addition, to solve the paradox, we elaborated a new „spatial” CPP test modifying the traditional CPP paradigm. In the latter the animals can learn the location of the conditioning place in two ways^23^: 1. recognizing some proximal cues of the conditioning place, when these cues become incentive, they can exert a positive magnetic effect (they are approached by the animals^24^) on the animal; 2. recognizing the spatial location of the place using external cues, so the cue itself does not become incentive or aversive, it can only help the spatial orientation of the animal. The two learning strategies do not exclude each other, both can be applied by the animal in the traditional CPP paradigm, consequently, perhaps, if one of these remains intact, then CPP can develop. In the new spatial version of the CPP test, however, the animals can learn to recognize the spatial location of the place only using external cues. If our hypothesis is true, then the intra-VP sulpiride- treated rats cannot learn the exact spatial location of the conditioning area in the spatial CPP test.

## Methods

### Animals and surgery

In the present experiments, 124 male Wistar rats weighting 280–320 g were housed individually. All animal experiments were conducted and all animals were cared for according to federal and local ethical guidelines, and the protocols were approved by the National Scientific Ethical Committee on Animal Experimentation of Hungary (BA02/2000-8/2012, BA02/2000-65/2017 and BA02/2000-64/2017, Pécs University, Medical School; Hungarian Government Decree, 40/2013. (II. 14.); NIH Guidelines, 1997; European Community Council Directive 86/609/EEC 1986, 2006; European Directive 2010/63/EU of the European Parliament). The present study is reported in accordance with ARRIVE guidelines.

Animals were kept in a temperature- and light-controlled room (21 ± 2 °C, 12:12 h light–dark cycle with lights on at 7:00 a.m.). Standard laboratory food pellets (CRLT/N Charles River Kft, Budapest, Hungary) and tap water were available for the animals ad libitum.

For the general anesthesia during the operation, intraperitoneal injection of a 4:1 ratio mixture of ketamine (Calypsol, Richter Gedeon, Hungary, 80 mg/kg body weight) and diazepam (Seduxen, Richter Gedeon, Hungary, 20 mg/kg body weight, 2 ml/kg b. w. of the mixture) were used. By means of the stereotaxic technique bilateral stainless steel guide tubes (22 gauge) were chronically implanted 0.5 mm above the VP (coordinates referring to the bregma: AP: − 0.26 mm, ML: 2.2 mm, DV: 7.1 mm from the surface of the dura) of the anesthetized rats according to the stereotaxic rat brain atlas of Paxinos and Watson^25^. The cannulae were fixed to the skull with self-polymerizing dental acrylic (Duracryl) anchored by 3 stainless steel screws. The guide tubes, when not being used for microinjection, were occluded with stainless steel obturators made of 27 gauge stainless steel wire. Drugs or vehicle were bilaterally microinjected through 27 gauge stainless steel microinjection tubes inserted into the guide cannulae. After the operation, the animals were allowed to have a 6-day long postoperative recovery before the experiments started, and during this period they were habituated to the experimenter.

### Drugs and microinjection protocol

The D2R antagonist sulpiride (Sigma-Aldrich Co.: (S)-(−)-Sulpiride, S7771) was bilaterally microinjected in three different doses: 0.1 μg, 1.0 μg, and 4.0 μg per side in 0.4 μl volume (0.73 mM, 7.32 mM, 29.29 mM, respectively). Controls received vehicle (0.4 μl). Thus, we had the 0.1D2anta, 1.0D2anta, 4.0D2anta and the control groups, respectively. Sulpiride was dissolved in 0.1 N HCl, and after the addition of phosphate buffer it was titrated with 0.1 N NaOH. Control animals received vehicle (veh.) in equal volume to that used for the drug injections. The solutions were kept at + 4 °C before their applications. All the mentioned doses are meant to be the dose per side values. Stainless steel microinjection tubes extended 0.5 mm below the tips of the implanted guide cannulae. The delivery cannula was attached to a 10 µl Hamilton microsyringe (Hamilton Co., Bonaduz, Switzerland) via polyethylene tubing (PE-10). All injections were delivered by a syringe pump in the volume of 0.4 µl (Cole Parmer, IITC, Life Sci. Instruments, California) over a 60 s interval. After accomplishing the microinjection, the cannulae were left in place for 60 s to allow diffusion into the surrounding tissue. Rats were gently held by hand during the injection procedure.

### Behavioral experiments

In the present experiments, all behavioral data were recorded, stored and evaluated by the Noldus EthoVision Basic system (Noldus Information Technology b.v., Wageningen, Netherlands). All the experiments were carried out on male Wistar rats in a sound- and climate-proof room (temperature: 22 ± 2 °C). Behavioral tests were performed during the daylight period between 08:00 and 18:00 h.

#### Morris water maze test

**Figure 1:**
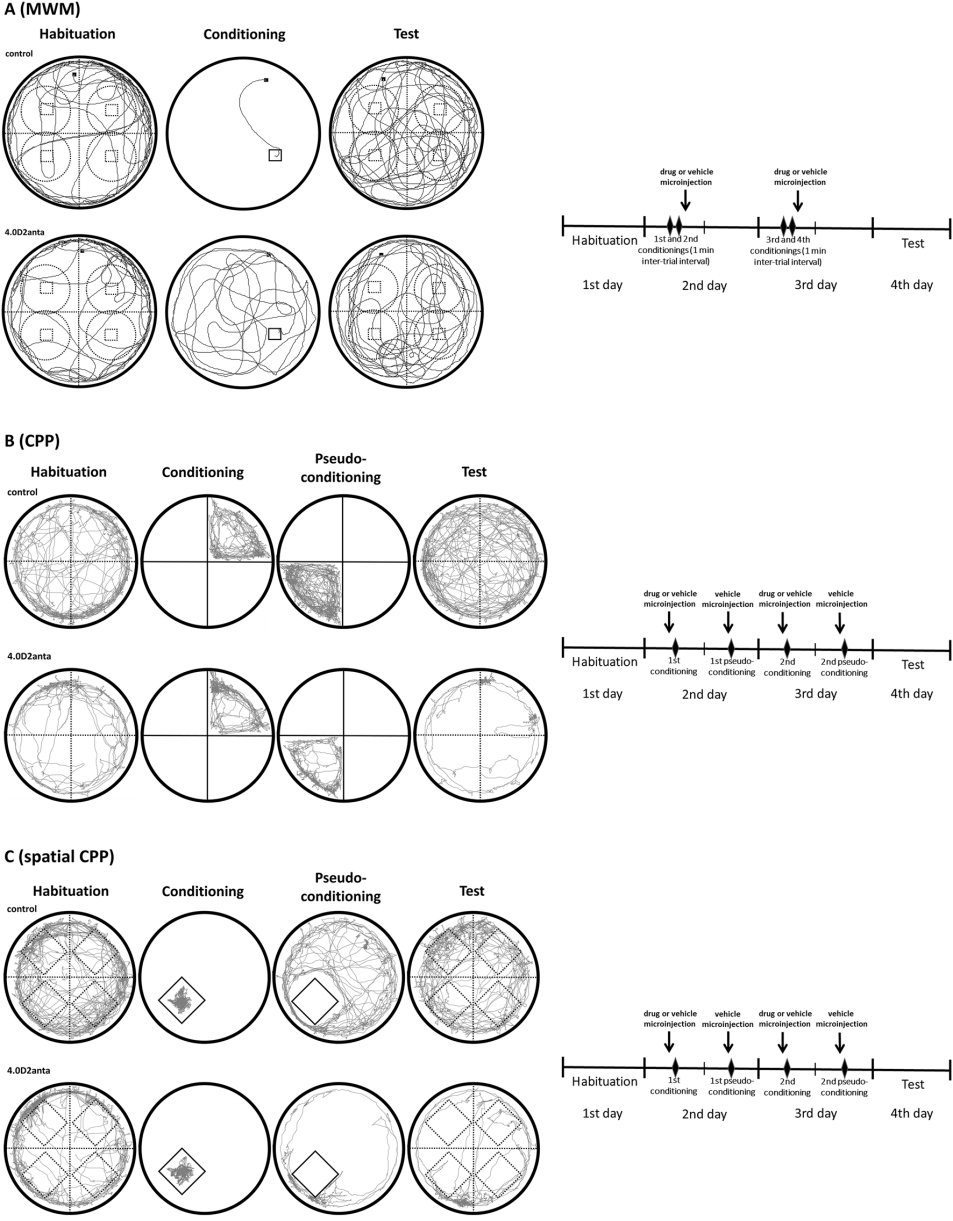
Representative example tracks of control and 4.0 µg sulpiride treated (4.0D2anta) animals in each paradigm within the sketch of the experimental apparatuses (on the left side) and diagrams illustrating the experimental schedule of each paradigm (on the right side). Solid lines in the sketch of the experimental paradigms demonstrate physical barriers (the boundaries of the apparatus or the plexiglass barriers in the traditional or spatial CPP) or the platform in MWM paradigm. Dashed lines indicate the boundaries of virtual spaces, i.e. the place of the platform or the virtual platforms and the surrounding area in MWM paradigm, the virtual quadrants in MWM, traditional CPP and spatial CPP paradigms, furthermore the place of the conditioning area and the virtual pseudo-conditioning area in spatial CPP paradigm. Panel (**A**) displays a schematic illustration of the MWM paradigm. Representative example tracks show that the 4.0 µg sulpiride treated animal found the platform with a longer latency compared to the control one during the conditioning trial. In the test trial both animals searched the platform in the central area of the apparatus, but the searching strategy of the control animal was more specific to the place of the platform (this latter was confirmed by the quantitative analysis). Panel (**B**) shows a schematic illustration of the traditional CPP paradigm. In the test trial it can be seen that the 4.0 µg sulpiride treated animal spent more time in the conditioning quadrant than in the other quadrants, and its locomotor activity decreased compared to the control animal. Panel (**C**) demonstrates a schematic illustration of the spatial CPP paradigm. The time spent in the conditioning area was not significantly changed in the case of either animal if we compare the habituation and the test trial. However, the 4.0 µg sulpiride treated animal spent more time in the neighborhood of the conditioning zone, thus in the virtual quadrant containing the real conditioning area compared to the other virtual quadrants. All this means that the 4.0 µg sulpiride treated animal could not learn the specific spatial location of the conditioning area.

The apparatus of the MWM test (Fig. 1A) was a circle pool (diameter = 1.5 m) filled with water (temperature: 23 ± 1 °C) and virtually divided into four quadrants. The square (10 × 10 cm) plexiglass platform was placed in one of the virtual quadrants (see below the description). The position of the platform was fixed throughout the experiments. The surface of the water was kept 2 cm above the platform and the water was colored with methylene blue to make the platform hidden for the animals. We applied the allocentric version of the MWM test^26–28^, where the rats had to find a hidden platform and they were placed into the water maze at randomly assigned but predetermined locations to avoid the egocentric orientation. The rats could swim for 180 s in the pool surrounded with external cues helping the orientation of the rats.

On the first day, a habituation trial was carried out, the platform was removed, and rats were allowed to swim freely. On the second and third day the platform was placed into the pool and two-two swimming trials were performed (conditioning 1st and 2nd, and conditioning 3rd and 4th, respectively). On both conditioning days, trials were carried out with 1 min inter-trial interval, and immediately after the two trials, sulpiride or vehicle was microinjected. On the fourth day the platform was removed, and a test trial was performed.

In the habituation and test trials the latency to find the place of the platform was measured. In each trial the distance moved, and the time spent in the inner zone (radius = 60 cm, surrounding the center of the pool) of the pool were also measured. In the habituation and test trials, we monitored the time spent in the zone (radius = 25 cm) surrounding the place of the platform. Moreover, in the other virtual quadrants of the pool, in the position identical to the place of the real platform, virtual platforms were assigned, and the time spent in the zones surrounding these virtual platforms was also monitored and then averaged (this was the time spent in the zone surrounding the virtual platform). When the time spent in the inner zone and the surrounding zones were calculated, the results of the habituation trial were subtracted from those of the test trial.

#### Conditioned place preference

In the traditional CPP test animals that spend more time in the drug-associated compartment are considered to exhibit conditioned place preference. The traditional CPP test (Fig. 1B) was carried out in a circular open field-based apparatus (radius = 42.5 cm)^29,30^. The floor and the walls of the equipment were gray-colored, the floor was divided into four equal quadrants by black lines. The room was dimly lit by a 40 W bulb. The area of the apparatus was divided into four virtual quadrants.

Both external and internal/proximal visual cues helped the orientation and learning of the animals^31^. The CPP procedure was performed in 4 days, consisting of one habituation (1st day), two conditioning trials (morning) and two pseudo-conditioning trials (afternoon, when the animals were treated only with the vehicle solution) (2nd–3rd day), and one test trial (4th day), each session lasted for 900 s (15 min). In the habituation and test trials, animals placed into the apparatus were allowed to move freely in the entire area of the apparatus. The time spent by the rats in each of the four quadrants was measured. In the conditioning trials the quadrants were separated by a plexiglass barrier, and the sulpiride solutions or vehicle were microinjected 5 min before the animals were put into the conditioning quadrant. In the pseudo-conditioning trials the rats were placed into the quadrant opposite to the conditioning quadrant. The pseudo-conditioning trials can help the animals to discriminate that not the whole area of the apparatus should be associated with the potential rewarding effect of the drug, but only the conditioning area. To demonstrate the change in the place preference of the rats, the time spent in the conditioning quadrant during the habituation was subtracted from those of the test trial. In each trial, the distance moved, and the time spent in the inner zone (radius = 30 cm) of the apparatus were measured.

#### Spatial conditioned place preference test

The spatial CPP test, elaborated in our laboratory, is a modified version of the traditional open field-based CPP paradigm, to investigate reward (or aversive) processes which requires spatial learning capabilities of the animals. The spatial CPP test (Fig. 1C) was carried out in an apparatus similar to that used in the traditional CPP paradigm. However, an important difference between the two paradigms was during the conditioning days. In the traditional CPP paradigm the animals were confined to one quadrant (called conditioning quadrant), which could be recognized by the animals based on external and proximal/internal cues (the internal cues can be approached directly by the animals, so it can be enough to memorize the specific cue(s) to recognize the conditioning quadrant in the traditional CPP paradigm). In contrast, in the spatial CPP paradigm the animals were placed in a 20 × 20 × 30 cm square-based plexiglass column (conditioning area), which could be recognized only based on its spatial location, similarly to the platform of the MWM paradigm. The schedule of the modified CPP was the same as that of the traditional CPP test. The area of the apparatus was divided into four virtual quadrants. In the conditioning and pseudoconditioning trials a square-based plexiglass column (hereafter the zone/area demarcated by this plexiglass will be called as conditioning area) was placed in one of the four quadrants (named ‘quadrant containing the conditioning area’) in an excentric position and not too close to the wall of the apparatus. The same visual cues used in the traditional CPP test were applied, but all of them could be considered as external cues since they were out of the conditioning area. This means that the animals were compelled to find the place of the conditioning only based on its spatial location and apply their spatial learning capabilities during the conditioning process.

In the habituation and test trials, the rats were allowed to move freely in the whole area of the apparatus. In the conditioning trials the animals were placed directly into the conditioning area 5 min after the microinjection. In the pseudo-conditioning trials, rats were allowed to move freely in the area external to the conditioning area (pseudo-conditioning area).

The time spent in the conditioning area was monitored in both the habituation and test trials. In addition, the time spent in the areas (3 areas) corresponding to the conditioning area but located in the quadrants not containing the conditioning area was also measured, and then averaged (this is the time spent in the virtual pseudo-conditioning area). The results of the habituation trial were subtracted from those of the test trial in the case of each animal. To observe the presence of the spatial CPP, the time spent in the conditioning area and the virtual pseudo-conditioning area was compared to each other. In addition to this new parameter, all the parameters determined in the case of the traditional CPP were analyzed.

### Histology and statistical analysis

At the end of the experiments, rats received an overdose of urethane (i. p. injection of 40% urethane solution, in a dose of 1.4 g/kg bw.) and were transcardially perfused with isotonic saline (slow infusion rate: 500 ml/20 min) followed by 10% formaldehyde solution (slow infusion rate: 500 ml/20 min). Their brains were removed and a week after fixation, brains were frozen, cut into 40 µm serial sections, and stained with Cresyl-violet. Microinjection sites were reconstructed according to the rat brain stereotaxic atlas^25^, the cannula tracks and the tip positions were determined on the basis of the existence of debris of the destroyed elements and the extent of a moderate glial proliferation. Only data from animals with correctly placed cannulae were analyzed. Behavioral data of animals with the incorrect and diverse placements were not enough to draw far-reaching conclusions. For statistical analysis one-way and mixed-ANOVA was applied and followed by Bonferroni post hoc test. Statistical significance was established at *p* < 0.05.

## Results

### Histology

**Figure 2:**
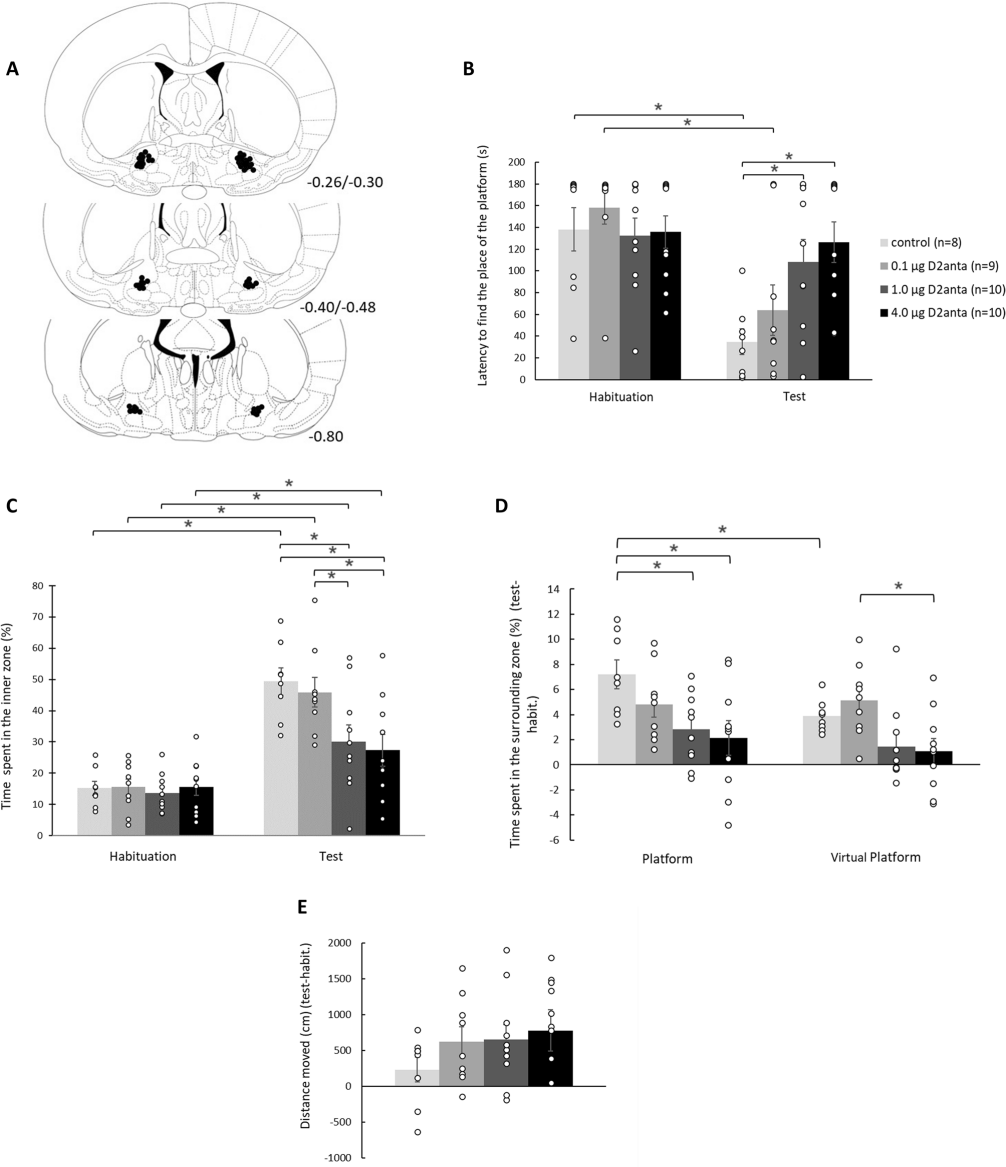
Effect of VP-sulpiride treatment on spatial learning in MWM test. Intra-VP sulpiride dose-dependently impairs learning processes, especially spatial learning. The intra-VP 1.0 µg and the 4.0 µg sulpiride completely impair spatial learning, but also the other, swimming-away strategy is impaired in their case. Panel (**A**) displays a schematic illustration of correct bilateral guide cannulae placement in the VP as shown in coronal sections of the rat brain taken from the Paxinos and Watson’s atlas^25^. The numbers beside refer to the anterior–posterior distance from the bregma in mm. Panel (**B**) shows the latencies (sec) to find the place of the platform, while panel (**C**) demonstrates the time spent (%) in the inner zone (r = 60) of the apparatus in the habituation and test trials. Panel (**D**) displays the time spent (%) in the zone surrounding the place of the platform and the virtual platform (test-habituation), to reveal the specific spatial learning. Panel (**E**) shows the distance moved by the animals (cm, test-habituation). Columns represent the averages ± the standard error of the mean (S.E.M.) in all panels, results of the individual subjects (represented as black circles) are added to the bar graphs. *p < 0.05 indicates significant differences. For further explanation, see the text.

**Figure 3:**
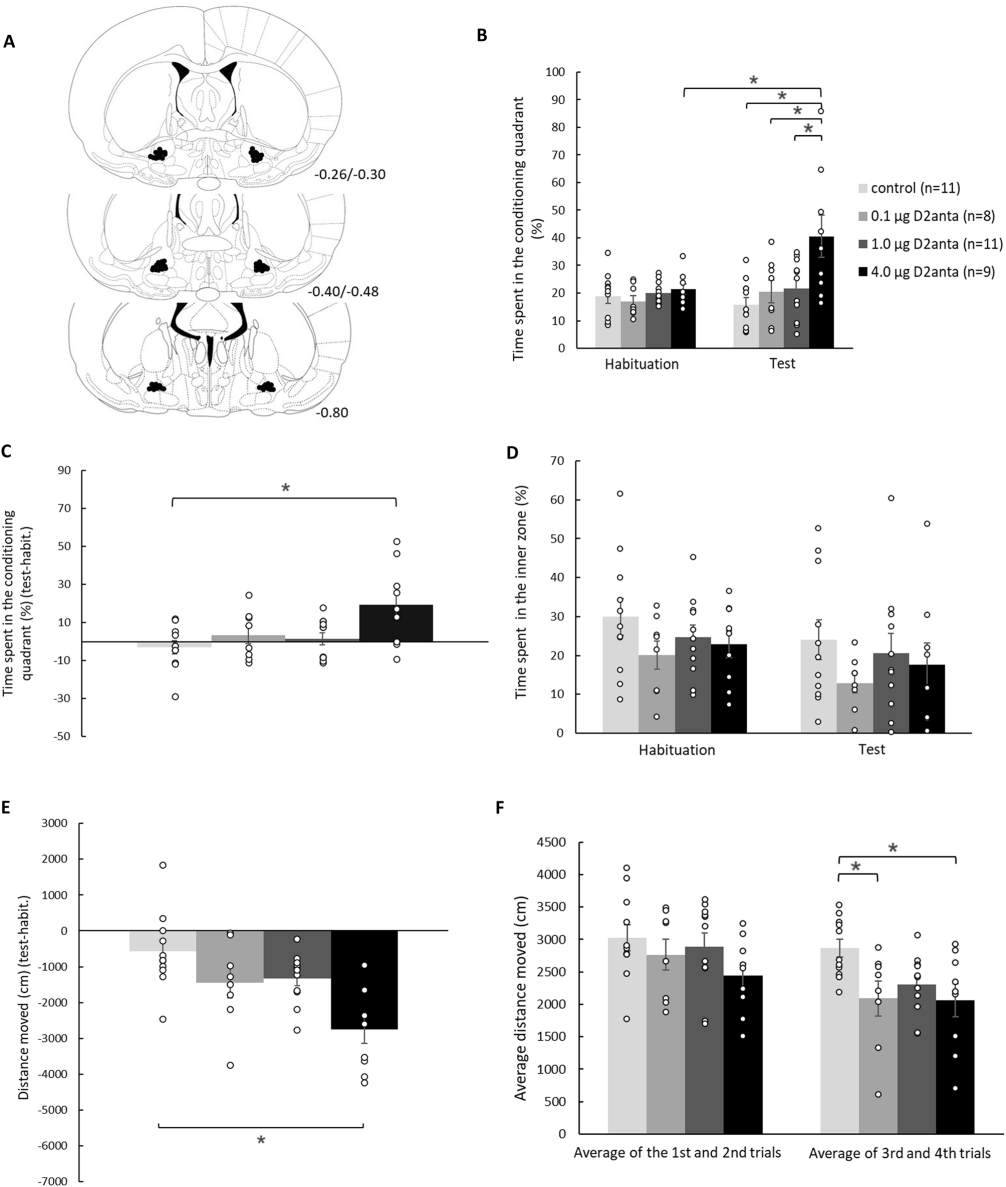
Dose-dependent rewarding effect of intra-VP sulpiride treatment in traditional CPP paradigm. The largest dose (4.0 µg) of the intra-VP sulpiride induced place preference. Furthermore, sulpiride microinjected into the VP, dose-dependently decreased locomotor activity of the animals. This was a gradual and durable effect. Panel (**A**) displays a schematic illustration of correct bilateral guide cannulae placement in the VP as shown in coronal sections of the rat brain taken from the Paxinos and Watson’s atlas^25^. The numbers beside refer to the anterior–posterior distance from the bregma in mm. Panel (**B**) shows the time spent (%) in the conditioning quadrant in the habituation and test trials. Panel (**C**) displays the time spent (%) in the conditioning quadrant subtracting the results of the test and the habituation trials in the case of each animal. The time spent (%) in the inner zone (r = 30) can be seen in panel (**D**), while panel (**E**) shows the distance moved by the animals (cm, test-habituation). Panel (**F**) demonstrates the average distance moved (cm) of the two conditioning trials within each conditioning day. Columns represent the averages ± the standard error of the mean (S.E.M.) in all panels, results of the individual subjects (represented as black circles) are added to the bar graphs. *p < 0.05 indicates significant differences. For further explanation, see the text.

**Figure 4:**
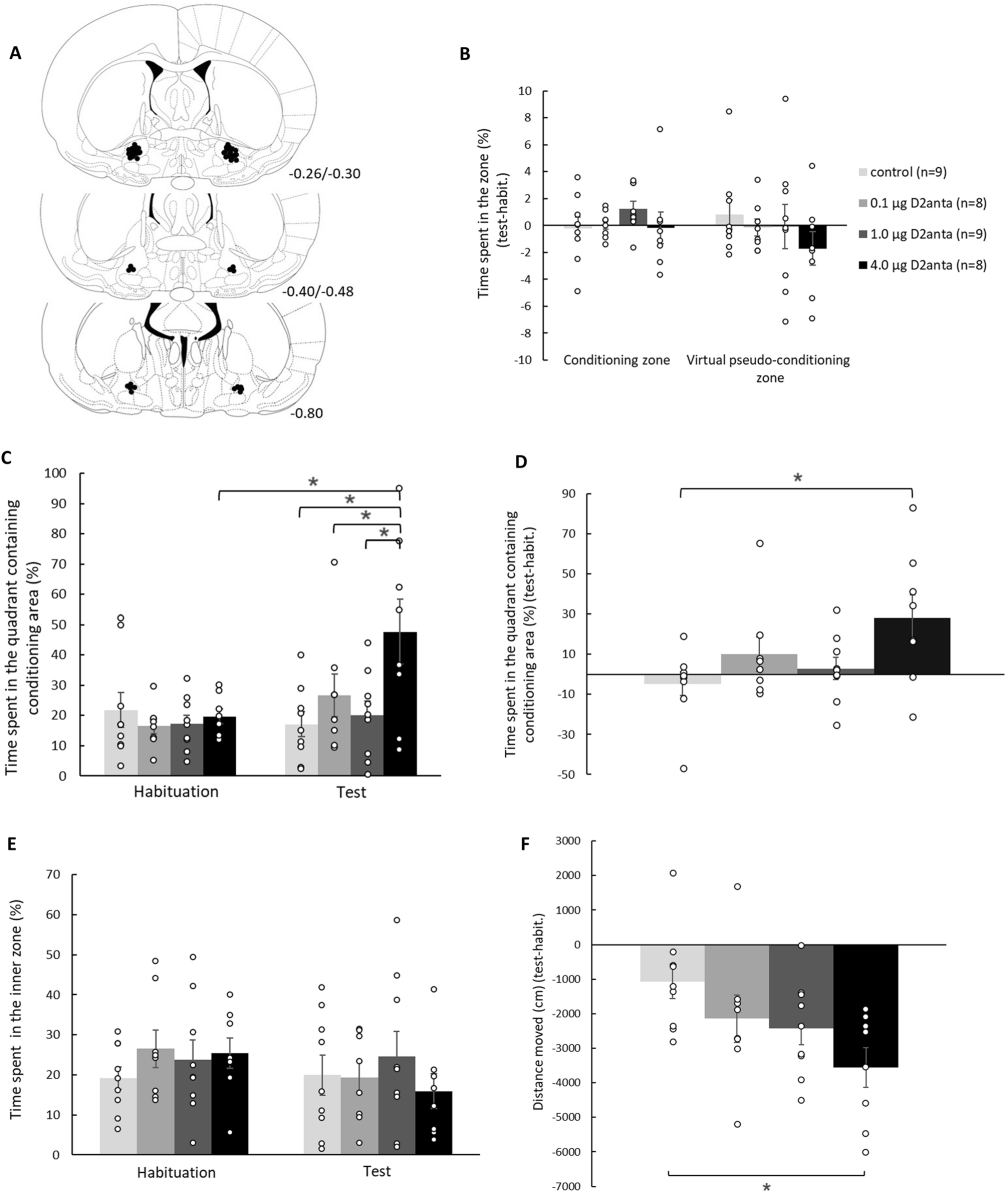
Dose-dependent rewarding effect of intra-VP sulpiride treatment in spatial CPP paradigm. The animals cannot associate the rewarding effect of the drug with the area that can be recognized only based on its spatial location (conditioning area), but they associated this positive “magnetic” effect with the quadrant containing the conditioning area (conditioning quadrant). This latter does not require the hippocampus-dependent spatial orientation and learning. Similar to the traditional CPP paradigm, intra-VP sulpiride microinjection evoked a dose-dependent reduction in locomotor activity of the animals. Panel (**A**) displays a schematic illustration of correct bilateral guide cannulae placement in the VP as shown in coronal sections of the rat brain taken from the Paxinos and Watson’s atlas^25^. The numbers beside refer to the anterior–posterior distance from the bregma in mm. Panel (**B**) shows the time spent (%) in the conditioning area (the squared based area, where the animals were confined during the conditionings) and virtual conditioning areas (test-habituation). Panel (**C**) and Panel (**D**) demonstrate the time spent (%) in the conditioning quadrant (containing the real conditioning area) in the habituation and test trials, in the latter case we calculated the difference between the two trials in case of each animal. In panel (**E**), the time spent (%) in the inner zone (r = 30) can be seen. Panel (**F**) shows the distance moved by the animals (cm, test-habituation). Columns represent the averages ± the standard error of the mean (S.E.M.) in all panels, results of the individual subjects (represented as black circles) are added to the bar graphs. *p < 0.05 indicates significant differences. For further explanation, see the text.

The histological examination showed that the guide cannulae were precisely and symmetrically located above the target area in 110 of the 124 animals. Schematic illustrations of cannula placements of the MWM, CPP, and spatial CPP experiments are shown in Figs. 2A, 3A, and 4A, respectively.

### Results of the Morris water maze test

In our present experiments MWM test was applied to reveal the effects of intra-VP sulpiride on spatial learning processes, to investigate how the various doses of sulpiride influence the different learning strategies.

The latency to find the place of the platform (Fig. 2B) was measured in the habituation and test trials, and mixed-ANOVA analysis revealed a significant trial effect (F(1,37) = 20.059; *p* = 0.001) and treatment*trial interaction (F(3,37) = 3.434; *p* = 0.027), however, treatment effect was not significant (F(3,37) = 2.729; *p* = 0.058). Bonferroni test demonstrated that in the habituation trial there was no difference among the groups, while in the test trial the platform finding latency of groups was significantly different, the controls found significantly faster the platform compared to the 1.0D2anta and 4.0D2anta groups (*p* = 0.026, *p* = 0.002, respectively). The pairwise comparison also showed a significant difference between the two trials: the control and the 0.1D2anta groups found the place of the platform in a significantly shorter time in the test than in the habituation trial (*p* = 0.001 in both cases).

The time spent in the inner zone (r = 60 cm) (Fig. 2C) of the apparatus was also monitored. Mixed-ANOVA revealed that there was a significant treatment effect (F(3,37) = 4.742; *p* = 0.007), trial effect (F(1,37) = 77.115; *p* = 0.001) and a treatment*trial interaction (F(3,37) = 4.130; *p* = 0.013). Bonferroni test showed significant differences among the groups only in the test trial. Pairwise comparison demonstrated that in the test trial the 1.0D2anta and 4.0D2anta groups spent significantly less time in the inner zone compared to the control (*p* = 0.003, *p* = 0.001, respectively), and 0.1D2anta groups (*p* = 0.019, *p* = 0.003, respectively). Furthermore, pairwise comparison also detected significant difference within the groups comparing the results of the habituation and the test trials: every group spent significantly more time in the inner zone in the test than in the habituation trial (control, 0.1D2anta, 1.0D2anta and 4.0D2anta groups; *p* = 0.001, *p* = 0.001, *p* = 0.002, *p* = 0.026, respectively).

The time spent (%) in the zone surrounding (r = 25 cm) (Fig. 2D) the platform and the virtual platform was measured, and the difference between the test and habituation trials was calculated to reveal the spatial specificity of learning. The mixed-ANOVA analysis showed a significant treatment (F(3,37) = 4.659; *p* = 0.007) and a location/position effect (F(1,37) = 13.720; *p* = 0.001), moreover a significant treatment*location interaction (F(3,37) = 3.872; *p* = 0.017). The pairwise comparison revealed that the 1.0D2anta and 4.0D2anta groups spent significantly less time in the area surrounding the platform compared to the controls (*p* = 0.020, *p* = 0.005, respectively). In the zone surrounding the virtual platform only the 4.0D2anta group spent significantly less time than the 0.1D2anta group (*p* = 0.028). Comparing the time spent in the zone surrounding the platform and virtual platform within each group, Bonferroni test revealed that only the controls spent significantly more time in the zone of the platform than in the zone of the virtual platform (*p* = 0.001).

The locomotor activity of the animals (Fig. 2E) in the habituation and test trials was also measured and the difference in the distance moved between the test and habituation trials was calculated. The one-way ANOVA analysis showed no difference among the groups (F(3,36) = 0.983; *p* = 0.413).

Results of the Fig. 2 show that the intra-VP sulpiride dose-dependently impairs learning processes in MWM paradigm (see Fig. 2B). This effect, mainly, can be due to the impairment of specific spatial learning (see Fig. 2D). All groups learned that the platform is in the inner zone (see Fig. 2C), though the largest doses of sulpiride impaired this learning process as well. Locomotor activity was not significantly influenced by the intra-VP sulpiride treatment (see Fig. 2E).

### Results of the conditioned place preference test

In the traditional CPP paradigm our goal was to reveal the potential rewarding or even aversive effect of the intra-VP sulpiride.

Considering the time spent in the conditioning quadrant (%) parameter (Fig. 3B) in the habituation and test trials, the mixed-ANOVA analysis revealed a significant treatment (F(3,39) = 5.647; *p* = 0.003), a significant trial effect (F(1,39) = 5.671; *p* = 0.022) and a significant treatment*trial interaction (F(3,39) = 4.746; *p* = 0.006). Bonferroni test showed that in the habituation trial there was no difference among the groups, however in the test trial the 4.0D2anta group spent significantly higher percentage of time in the conditioning quadrant than all the other groups (*p* = 0.001 in all cases). Furthermore, the time spent in the conditioning quadrant was significantly increased in the test trial compared to the habituation trial in the case of the 4.0D2anta group (*p* = 0.001).

The difference in time spent in the conditioning quadrant between the test trial and habituation trial (test-habituation, %) (Fig. 3C) was analyzed by means of one-way ANOVA, and a significant difference was found among the groups (F(3,38) = 4.259; *p* = 0.012). Bonferroni test demonstrated that the 4.0D2anta group spent significantly more time in the conditioning quadrant compared to the controls (*p* = 0.010).

The time spent (%) in the inner zone (r = 30 cm) (Fig. 3D) of the apparatus in the habituation and test trials was also measured. The mixed-ANOVA analysis showed a significant trial effect (F(1,39) = 5.113; *p* = 0.029), but not a significant treatment effect (F(3,39) = 1.615; *p* = 0.201) or treatment*trial interaction (F(3,39) = 0.066; *p* = 0.978).

The locomotor activity of the animals was also monitored and the difference in the distance moved between the test and habituation trials was calculated (Fig. 3E). The one-way ANOVA analysis revealed a significant difference among the groups (F(3,38) = 3.802; *p* = 0.018). The Bonferroni test showed that the 4.0D2anta group moved less compared to the controls (*p* = 0.011).

Analyzing the distance moved during the conditioning days the average of the two trials within each day was calculated (Fig. 3F). The mixed-ANOVA analysis demonstrated a significant trial effect (F(1,39) = 48.588; *p* = 0.001), a non-significant treatment effect (F(3,39) = 2.702; *p* = 0.059) and a significant treatment*trial interaction (F(3,39) = 3.331; *p* = 0.029). Bonferroni test showed that the average distance moved by the 0.1D2anta and 4.0D2anta treated groups was significantly decreased compared to the controls (*p* = 0.048, *p* = 0.028, respectively) on the second conditioning day.

Results of the Fig. 3 demonstrate that the largest dose of intra-VP sulpiride induces place preference (see Fig. 3B and C). Furthermore, intra-VP sulpiride dose-dependently and gradually decreases the distance moved by the animals (see Fig. 3F) leading to a reduced locomotion even 24 h after the last treatment (see Fig. 3E).

### Results of the spatial conditioned place preference test

The spatial CPP test was developed in our laboratory to dissociate drugs’ rewarding effect and their potential damaging effect on spatial learning processes. This test can show whether the animals can associate the rewarding effect with an area which can be recognized only on its spatial location.

In the spatial CPP test, the time spent (%) in the conditioning area and virtual conditioning area (Fig. 4B) was measured and compared to each other using mixed-ANOVA statistical analysis. The analysis revealed that treatment effect (F(3,34) = 0.915; *p* = 0.444), location/position effect (F(1,34) = 0.573; *p* = 0.454), and treatment*position interaction (F(3,34) = 0.767; *p* = 0.521) were not significant.

However, when the time spent (%) in the quadrant containing the conditioning area was analyzed in the habituation and test trials (Fig. 4C), mixed-ANOVA revealed a significant treatment (F(3,34) = 3.587; *p* = 0.024) and trial effect (F(1,34) = 5.815; *p* = 0.021), moreover a significant treatment*trial interaction (F(3,34) = 3.399; *p* = 0.029). Bonferroni test showed a significant difference among the 4.0D2anta group and all the other groups (control, 0.1D2anta and 1.0D2anta groups, *p* = 0.001, *p* = 0.047, *p* = 0.002, respectively) only in the test trial. Furthermore, the time spent in the quadrant containing the conditioning area was significantly increased in the test trial compared to the habituation trial in the case of the 4.0D2anta group (*p* = 0.001).

In addition, the difference in time spent in the conditioning quadrant between the test and habituation trials (test-habituation, %) (Fig. 4D) was also analyzed. One-way ANOVA showed a significant difference among the groups (F(3,33) = 2.999; *p* = 0.046). Bonferroni test demonstrated that the 4.0D2anta group spent significantly more time in the conditioning quadrant compared to the controls (*p* = 0.044).

The time spent in the inner zone (r = 30 cm) (Fig. 4E) of the apparatus was also measured both in the habituation and test trials. The mixed-ANOVA analysis showed no significant treatment (F(3,34) = 0.406; *p* = 0.705) and trial (F(1,34) = 1.822; *p* = 0.186) effects, as well as no significant treatment*trial interaction (F(3,34) = 0.890; *p* = 0.456).

Regarding the difference in the distance moved between the test and habituation trials (Fig. 4F) one-way ANOVA analysis detected a significant difference among the groups (F(3,33) = 3.406; *p* = 0.030). The Bonferroni test showed that the 4.0D2anta group moved significantly less compared to the controls (*p* = 0.021).

Results of the Fig. 4 revealed that the largest dose of intra-VP sulpiride could not induce place preference for the conditioning area (see Fig. 4B), if it could be recognized only based on its spatial location. Nevertheless, place preference was induced by the largest dose of sulpiride for the quadrant which contained the conditioning area (see Fig. 4C and D). This indicate that the reward related learning process was inaccurate, it was not spatial learning, the animals cannot find the exact place where they got the rewarding treatment. Dose-dependent locomotor reducing effect of the intra-VP sulpiride was confirmed in this paradigm as well (see Fig. 4F).

## Discussion

In the present experiments, our main question was how the sulpiride microinjected into the VP can influence motivational and learning processes in rats. In the allocentric MWM test^26,27,32^, animals can find the invisible platform in two ways: they can learn its exact spatial location, and/or that they have to swim away from the edge of the pool to the inner zone, where they can find the platform with higher probability. Our results demonstrated that only the controls spent significantly more time in the zone surrounding the place of the platform compared to that of the virtual platform revealing that only the controls could learn the specific spatial location of it. Nevertheless, all groups spent more time in the inner zone of the pool in the test compared to the habituation trial, which means they learned that the platform is in the inner zone, though the largest doses of sulpiride also impaired this learning process. Even so, the largest dose of sulpiride induced CPP in the traditional CPP paradigm. How is it possible that the same dose of sulpiride reduced necessary learning skills^22,23^ in one paradigm, while it seemed to spare learning in the other? To solve this discrepancy, we elaborated the spatial version of the CPP paradigm. Applying this, we could observe that the rats could not associate the rewarding effect of the drug with that area which could be recognized only based on its spatial location. Thus, we could solve the apparent paradox: the formation of the traditional CPP requires only the partial intactness of learning processes, the spatial learning skills are not essential for it. Interestingly, in the spatial CPP paradigm, in the test trial, the largest dose sulpiride treated animals did not spend more time in the conditioning area, but in the quadrant containing the conditioning area. Theoretically, in the pseudo-conditioning trials, extinction takes place for the area external to the conditioning zone, helping the learning of the animals to discriminate the treatment place and the other parts of the apparatus. We suppose, and the results of the MWM paradigm support the plausible explanation, that also the 4.0 µg sulpiride treated rats learned that in the spatial CPP apparatus something was rewarding, but their association was inaccurate, they could not identify the exact place where they were treated. Consequently, this led to that these rats spent more time in the area close to the conditioning area, however they could locate the place of the treatment accurately in the space.

Data obtained from the traditional CPP test are considered as being measures of the rewarding, reinforcing effect of drugs^31^. According to the central theory of reinforcement, reinforcer agents facilitate memory and learning processes^33^. Our present results demonstrated the existence of an important counterexample when a reinforcer agent is not a universal enhancer of learning.

It is well-known that the hippocampus, strongly involved in the control of spatial learning^5,27,34^, regulates the VTA DAergic population activity via the NAC-VP axis^35^, which, doing so, modulates the formation of long-term memory^6^. Furthermore, it has been shown that the 3-day systemic administration of the D2R antagonists induces synaptic degeneration likely involving inhibitory synapses exclusively in the VP^36^. This synaptic degeneration perhaps can be regarded as an endpoint of an extreme synaptic weakening induced by the D2R antagonists, but certainly diminishes the effect of the affected input. The main inhibitory input to the VP originates in the NAC, supplying it with rich GABAergic innervation^37^. Based on these findings we can hypothesize that the intra-VP sulpiride probably weakened gradually and permanently the GABAergic fibers originating in the NAC, consequently leading to an enhanced inhibition of the VTA by the VP and resulting in the impairment of learning processes.

The DA, and mainly the DA released in the NAC, is considered as a rewarding neurotransmitter^38^. In the NAC shell region, the D2R agonist quinpirole induces CPP ^39^, but interestingly, the sulpiride cannot induce CPA^40^. In the medial septum, blocking D2Rs leads to CPA and it also suppresses locomotor activity^41^. In contrast, in the lateral hypothalamus, sulpiride has rewarding effect and enhances locomotor activity^42^. It has been demonstrated that these latter effects are realized via the VTA activation and the resulting NAC D2R activation^42,43^. Recently, we have shown that the largest dose of the D2R agonist quinpirole in the VP leads to CPA and reduces the VTA DAergic activity^14^. These facts make it plausible that also the rewarding effect of intra-VP sulpiride prevails via the VTA DAergic activation. Indeed, in a recent paper, it has been shown that the activation of the D3R expressing (D3R +) VP neurons facilitates VTA DAergic activity and drives DA release in the NAC evoking real-time place preference^44^. It is well-known that the DA can inhibit neurons via the D2Rs^45^. Thus, it can be assumed that the sulpiride, microinjected into the VP, can eliminate the inhibitory DA tone on these D3R + VP neurons, and consequently, it can increase the VTA DAergic activity, releasing DA in the NAC shell region and inducing CPP. Another alternative explanation can be that the acute effect is presynaptic as well, affecting the GABAergic fibers originating in the NAC. This is supported by the work of Napier who demonstrated that the quinpirole mostly increases the VP neuronal activity and sulpiride can eliminate this effect^46^. Thus, we can suppose that sulpiride microinjected into the VP can eliminate the effect of the endogenous DA on the presynaptic D2Rs, disinhibiting the NAC GABAergic fibers, and so inhibiting VP neurons. As a consequence, VTA can be released from the VP inhibition, and this can lead to the CPP induction.

In addition to its rewarding effect, DA in the NAC increases locomotor activity as well^47,48^. If we suppose that the intra-VP sulpiride did induce CPP via the VTA-NAC axis, then also the locomotor activity should have been increased. Nevertheless, the opposite can be seen, the sulpiride gradually decreased the locomotor activity. This effect was relatively durable because it could be observed also one day after the last sulpiride treatment. All this means, that the locomotor effect cannot be an acute effect of the intra-VP sulpiride. Moreover, our results suggest that it is likely not to be a simple motor effect. In the MWM paradigm, when the sulpiride was administered after the trials, locomotion was not influenced by the drug, demonstrating that the gradually decreasing locomotion was associated with the treatment-environment. It has been shown that the VP is a central element of the circuit, which plays an important role in the initiation of adaptive behavioral responses to environmental stimuli^49^ and novelty^50^. If so, then we can assume that the animals’ reactivity to the environment is reduced by the intra-VP sulpiride treatment, and probably a faster habituation can be observed compared to the controls.

The synaptic degeneration, induced by the intra-VP D2R antagonists is not an acute, but a gradually developing effect. It has been shown that stimulation of D2R-expressing medium spiny neurons in the NAC increases VTA DAergic population activity via the VP^51^. If we hypothesize that the synaptic degeneration involves these DAergic fibers, then we get a consistent explanation for our present findings concerning the spatial learning impairing and the locomotion reducing effect of intra-VP sulpiride. Following this thread, we can state more. Yao et al. found that D2R expressing neurons of the dorsomedial NAC shell region innervate glutamatergic neurons of the VP, whereas D2R expressing neurons of the ventral NAC shell region terminate on VP GABAergic cells^52^. It is known that the stimulation of the VP glutamatergic cells decreases VTA DAergic neuron activity^53^, while that of the VP GABAergic cells rather increases it^54–56^. In this respect, we can better define the NAC GABAergic fibers affected by the intra-VP sulpiride treatment: likely they originate in the dorsomedial NAC shell region. This is also supported by the fact that the D2R expressing neurons of this NAC region receive input mainly from the hippocampus, compared to the other shell regions^52^.

Potential limitation of the present study can be that the sulpiride, in high concentrations, can have non-specific effects on other receptors, mainly on serotonin receptors. Serotonin receptors (5-HT1B) in the VP may be critically involved in the antidepressant action of ketamine^57^. Cocaine inhibits GABA transmission from the NAC to the VP involving elevation of serotonin and activation of 5-HT1B receptors^58^. The activation of 5-HT2C receptors in the VP leads to a reduced locomotor activity^59^. Based on these findings, we cannot exclude completely the possibility that the sulpiride affected serotonin receptors in the VP, however, this seems to be not too probably, since in this case we could observe increased locomotor activity, supposing that the sulpiride blocked the effect of serotonin on its 5-HT2C receptors. Nevertheless, we do not have enough data concerning the role of VP serotonin in motivation and learning processes, therefore this issue requires further investigations.

In summary, sulpiride, microinjected into the VP, induces CPP in a dose-dependent fashion, presumably via the prompt presynaptic and/or postsynaptic mechanisms, indirectly activating the VTA DAergic neurons. This is an acute effect, which is necessary to establish CPP since it requires the temporal overlap between the effect of the drug and the conditioning environment^22,23^. In contrast, the spatial learning impairment and the locomotion decreasing effect probably can be due to the degeneration of the D2R expressing GABAergic fibers originating in the NAC shell region, leading to a decreased VTA DAergic population activity. This is a gradually developing, long-run effect, and it might have a great clinical relevance since one of the main etiological factors underlying the positive symptoms of schizophrenia is the overactivity of the VTA DAergic neurons^60^. In this way, we have revealed a potential mechanism for how the D2R antagonist treatment can exert its effect in the limbic system.

## Data Availability

The datasets generated during and/or analysed during the current study are available from the corresponding author on reasonable request.
